# Factors related to amblyopia in congenital ptosis after frontalis sling surgery

**DOI:** 10.1186/s12886-018-0962-4

**Published:** 2018-11-21

**Authors:** Youn-Shen Bee, Pei-Jhen Tsai, Muh-Chiou Lin, Ming-Ying Chu

**Affiliations:** 1 Department of Ophthalmology, Kaohsiung Veterans General Hospital, 386 Ta-Chung 1st RD, Kaohsiung, 81346 Taiwan; 2 Yuh-Ing Junior College of Health Care and Management, Kaohsiung, Taiwan; 3 National Defense Medical Center, Taipei, Taiwan

**Keywords:** Congenital ptosis, Frontalis sling suspension, Amblyopia, Mersilene mesh, Silicone rode, Polytetrafluoroethylene

## Abstract

**Background:**

Amblyopia is a main concern in children undergoing frontalis sling surgery for repairing congenital ptosis. This study aimed to evaluate factors related to amblyopia in children undergoing frontalis sling surgery.

**Methods:**

IRB-approved retrospective review of children under the age of 12 who received frontalis sling surgery. Preoperative demographic data, strabismus, margin reflex distance 1 (MRD1), lid fissure height, sling type, refraction errors, surgical outcome and amblyopia were evaluated.

**Results:**

This study included 48 eyelid procedures performed in 38 patients. Median age was 4.0 years. Etiology was congenital ptosis in 42 eyes (87.5%) and blepharophimosis in 6 eyes (12.5%). Mersilene mesh was the sling material used in 36 eyes (75%), silicone in 6 eyes (12.5%), and polytetrafluoroethylene (PTFE) in 6 eyes (12.5%). Mean duration of follow-up was 27.8 ± 25.0 months (range, 3 to 128 months). Amblyopia was observed in 17 eyes (35.4%) at the final follow-up. Factors significantly associated with final amblyopia included blepharophimosis (*p* = 0.017), preoperative MRD1 ≤ − 1.0 mm (*p* = 0.038), preoperative lid fissure ≤4.5 mm (*p* = 0.035), preoperative anisometropia (spherical equivalent) (*p* = 0.011), and postoperative astigmatism (*p* = 0.026).

**Conclusions:**

Study results suggest that blepharophimosis, preoperative MRD1 ≤ − 1.0 mm, preoperative lid fissure ≤4.5 mm, preoperative anisometropia (spherical equivalent), and postoperative astigmatism are associated with amblyopia after frontalis sling surgery in patients with congenital ptosis.

## Background

The incidence of amblyopia has been reported to be higher among patients with childhood ptosis than in the general population [1–3]. This is likely the result of increased prevalence of eyelid occlusion of the visual axis, strabismus, and significant refractive error [4–7]. Mersilene mesh, silicone, and polytetrafluoroethylene (PTFE, Gore-Tex) have been successfully used in frontalis sling surgery for congenital ptosis [8–13]. This study was undertaken to evaluate factors related to amblyopia in children undergoing frontalis suspension surgery utilizing Mersilene mesh, silicone, or PTFE as the sling material.

## Methods

### Ethics

The study was approved by the Institutional Review Board of Kaohsiung Veterans General Hospital, allowing retrieval of patient charts and review of medical information. A waiver of consent was granted given the retrospective nature of the project and anonymous analysis of the data.

### Participants and procedures

Data from 48 eyelid procedures in 38 patients under the age of 12 who underwent frontalis sling surgery to correct congenital ptosis with poor levator function at Kaohsiung Veterans General Hospital between January 2005 and July 2014 were analyzed. Patients who had simple congenital ptosis or blepharophimosis without neurologic or traumatic pathology were included in this study. Patients with less than 3 months of follow-up were excluded.

All patients underwent an ophthalmic examination including orthoptic evaluation and cycloplegic refraction; however, preoperative refraction data were missing for some younger patients due to poor cooperation. Visual acuity was measured with age-appropriate methods, including the Snellen chart and Allen symbols. Anisometropia was defined as difference between the eyes in spherical equivalent or astigmatism in any meridian on cycloplegic refraction. Amblyopia was defined as best-corrected visual acuity within two Snellen lines compared with age-matched normal children and more than two Snellen lines of difference between eyes [7]. Patients diagnosed with amblyopia were treated according to individual patient condition. Frontalis sling surgery was performed with a pentagon incision and sling procedure. A sling suture was passed in a closed cerclage-type fashion through skin entry by way of a supra-lash or eyebrow incision. Mersilene mesh, silicone, or PTFE was used as the sling material. In cases using Mersilene mesh or PTFE of the single-loop design, two stab incision sites approximately 10–12 mm apart were marked above the lash line centered over the area of desired maximal elevation. In cases using silicone rod, one additional stab incision was made in the middle of the two previous incisions with double pentagon technique [14]. Another two stab incision sites were marked above the eyebrow, approximately in line with lateral and medial canthus; an additional stab incision site was made above the eyebrow in the middle of the previous eyebrow incisions. Sling material was tied together beneath the frontalis muscle layer in the middle incision site in all cases.

Data collected and analyzed included age, gender, diagnosis, presence of strabismus, presence of chin-up sign, margin reflex distance 1 (MRD1), lid fissure height, sling type, refraction error, MRD1 elevation, lid fissure elevation, surgical outcome, follow-up duration, and presence of amblyopia at the last visit. During follow-up, outcomes were categorized as good, moderate, or poor. A good outcome was defined as postoperative MRD1 ≥ 2 mm or bilateral nonsymmetric lid fissure ≤1 mm. A moderate outcome was defined as postoperative MRD1 < 2 mm or > 1 mm or bilateral non-symmetric lid fissure > 1 mm. A poor outcome was defined as postoperative MRD1 ≤ 1 mm or bilateral nonsymmetric ≥2 mm. Preoperative and postoperative photographs of each surgery were obtained using a digital camera (Nikon Inc., Tokyo, Japan) in examination rooms with equivalent lighting. Postoperative follow-up records and photographs were reviewed.

### Statistical analysis

Statistical analysis was performed using SPSS version 18.0 (SPSS Inc., Chicago, IL). Basic descriptive statistics were calculated using the data gathered and are reported as mean ± standard deviation or n (%) as appropriate. Differences between continuous outcome variables were established and putative factors were sought using the Mann-Whitney U-test and Student’s t-test as appropriate. Categorical data were examined using Pearson’s chi-square and Fisher’s exact test. All tests were two-tailed and a *p*-value ≤0.05 was considered statistically significant.

## Results

In total, data from 48 eyelid procedures in 38 patients were collected. Of the 38 patients, 27 had unilateral procedures and 11 had bilateral procedures. One female patient with bilateral ptosis experienced left ocular trauma with sling rupture 1 week after frontalis sling surgery, and this procedure was excluded. Twenty-four procedures were performed on the right side and 24 were on the left side. Age ranged from 1 to 12 years, with a mean age of 4.3 ± 2.4 years and median age of 4.0 years. Postoperative follow-up time ranged from 3 to 128 months, with a mean of 27.8 ± 25.0 months. Etiology was congenital ptosis in 42 eyes (87.5%) and blepharophimosis in 6 eyes (12.5%). Mersilene mesh was used as the sling material in 36 eyes (75%), silicone in 6 eyes (12.5%), and PTFE in 6 eyes (12.5%). Amblyopia was found in 23 eyes (47.9%) preoperatively. In 14 of these eyes, amblyopia was attributed to occlusion of the visual axis only, 9 eyes had high astigmatism or anisometropia, and 6 eyes exhibited a combined refractive and occlusive mechanism. Preoperative visual acuity (log MAR) was 0.28 ± 0.22 and postoperative visual acuity (log MAR) was 0.19 ± 0.21. Preoperative and postoperative refraction data are listed in Table 1. Due to poor cooperation of very young patients who were aged 1–2 years, preoperative refraction data were missing for 4 eyes. The demographics of the patients grouped by sling material used in surgery are shown in Table 1.

**Table 1:** Demographic data in total eyelids with different sling material

Factor	Total	Mersilene mesh	Silicone	PTFE (Gore-Tex)
	*n* = 48	*n* = 36	*n* = 6	*n* = 6
Age,years (mean ± SD)	4.28 ± 2.38	4.56 ± 2.49	3.15 ± 2.38	3.73 ± 1.42
Gender (male/female)	30/18 (62.5%/37.5%)	26/10 (72.2%/27.8%)	2/4 (33.3%/66.7%)	2/4 (33.3%/66.7%)
Side (Right/Left)	24/24 (50.0%/50.0%)	18/18 (50.0%/50.0%)	3/3 (50.0%/50.0%)	3/3 (50.0%/50.0%)
Lateral (uni/bil)	27/21 (56.2%/43.8%)	22/14 (61.1%/38.9%)	2/4 (33.3%/66.7%)	3/3 (50.0%/50.0%)
Diagnosis				
congenital	42 (87.5%)	33 (91.7%)	6 (100%)	3 (50.0%)
blepharophemosis	6 (12.5%)	3 (8.3%)	0 (0%)	3 (50.0%)
Follow period (months)	27.77 ± 24.99	28.61 ± 27.78	30.17 ± 8.70	20.33 ± 17.67
Pre-op MRD1^a^ (mm)	− 0.61 ± 1.37	− 0.68 ± 1.47	0.00 ± 0.95	− 0.83 ± 0.98
Pre-op Lid fissure (mm)	3.89 ± 1.06	3.86 ± 1.14	4.25 ± 0.76	3.67 ± 0.82
Pre-op chin up				
yes	31 (64.6%)	23 (63.9%)	4 (66.7%)	4 (66.7%)
no	17 (35.4%)	13 (36.1%)	2 (33.3%)	2 (33.3%)
Pre-op strabismus				
yes	10 (20.8%)	8 (22.2%)	2 (33.3%)	0 (0%)
no	38 (79.2%)	28 (77.8%)	4 (66.7%)	6 (100%)
Pre-op amblyopia				
yes	23 (47.92%)	17 (47.22%)	5 (83.33%)	1 (16.67%)
no	25 (52.08%)	19 (52.78%)	1 (16.67%)	5 (83.33%)
Pre-op log MAR	0.28 ± 0.22	0.28 ± 0.23	0.29 ± 0.15	0.28 ± 0.22
Post-op log MAR	0.19 ± 0.21	0.18 ± 0.20	0.21 ± 0.29	0.22 ± 0.23
Pre-op sphere^b^	−0.22 ± 2.01	0.01 ± 1.69	−0.79 ± 3.26	−1.05 ± 2.37
Post-op sphere^b^	0.35 ± 1.55	0.49 ± 1.70	−0.42 ± 0.74	0.25 ± 0.89
Pre-op astigmatism^b^	−1.47 ± 1.17	− 1.50 ± 1.29	−1.38 ± 0.80	−1.40 ± 0.76
Post-op astigmatism^b^	− 1.44 ± 1.32	−1.53 ± 1.32	−0.54 ± 0.68	−1.79 ± 1.62
Pre-op spherical equivalent^b^	−0.96 ± 1.97	−0.74 ± 1.56	−1.48 ± 3.45	−1.75 ± 2.41
Post-op spherical equivalent^b^	−0.37 ± 1.48	−0.27 ± 1.66	−0.69 ± 0.68	−0.65 ± 0.74
Pre-op anisometropia (astigmatism)^b^	1.03 ± 1.15	1.14 ± 1.27	0.79 ± 0.60	0.50 ± 0.47
Post-op anisometropia (astigmatism)^b^	0.94 ± 0.92	1.05 ± 1.03	0.75 ± 0.39	0.50 ± 0.16
Pre-op anisometropia (spherical equivalent)^b^	0.94 ± 0.92	0.68 ± 0.73	2.02 ± 0.98	1.35 ± 1.07
Post-op anisometropia (spherical equivalent)^b^	1.01 ± 1.10	1.07 ± 1.21	0.46 ± 0.06	1.25 ± 0.88

^a^marginal reflex distance 1

^b^Refraction unit = Diopter

Amblyopia was found in 17 eyes (35.4%) at the final follow-up. Eight eyes diagnosed with amblyopia preoperatively had normal visual acuity at final follow-up, and absence of both significant refraction error and strabismus besides lid drooping were common characteristics of these eyes. There was no significant association between age at operation and incidence of final amblyopia. At the final follow-up, 12 of 48 eyes (25%) with congenital ptosis and 5 of 6 eyes (83%) with blepharophimosis had amblyopia. Factors significantly associated with final amblyopia included blepharophimosis (*p* = 0.017), preoperative MRD1 (*p* = 0.018), preoperative MRD1 ≤ − 1.0 mm (*p* = 0.038), preoperative lid fissure (*p* < 0.001), preoperative lid fissure ≤4.5 mm (*p* = 0.035), and preoperative anisometropia (spherical equivalent) (*p* = 0.011) (Table 2). Presence of preoperative chin-up sign, strabismus, and preoperative amblyopia were not related to amblyopia at the final visit.

**Table 2:** Preoperative factors related to amblyopia in the final visit

Factor	No amblyopia^1^	Amblyopia^2^	*P* valve
	*n* = 31	*n* = 17	
Age, years (mean ± SD)	4.66 ± 2.41 (1–9)	3.57 ± 2.23 (1–8)	0.131
Gender (male/female)	22/9 (71.0%/29.0%)	8/9 (47.1%/52.9%)	0.093
Side (Right/Left)	17/14 (54.8%/45.2%)	7/10 (41.2%/58.8%)	0.547
Lateral (uni/bil)	20/11 (64.5%/35.5%)	7/10 (41.2%/58.8%)	0.140
Diagnosis			
congenital	30 (96.8%)	12 (70.6%)	0.017^a^
blepharophemosis	1 (3.2%)	5 (29.4%)	
Follow period (months)	28.68 ± 27.57 (3–128)	26.12 ± 20.12 (3–63)	0.738
Pre-op sphere^3^	−0.16 ± 1.79	−0.33 ± 2.42	0.426
Pre-op astigmatism^3^	− 1.27 ± 1.11	−1.83 ± 1.21	0.963
Pre-op spherical equivalent^3^	− 0.80 ± 1.87	−1.24 ± 2.16	0.457
Pre-op anisometropia (astigmatism)^3^	0.92 ± 0.83	1.20 ± 1.58	0.449
Pre-op anisometropia (spherical equivalent)^3^	0.68 ± 0.79	1.40 ± 0.98	0.001 ^b^
Pre-op MRD1 (mm)	−0.27 ± 1.21	− 1.24 ± 1.46	0.018^b^
pre-op MRD1 (mm) ≤ − 0.5	12 (38.7%)	11 (64.7%)	0.131
pre-op MRD1 (mm) ≤ −1.00	10 (32.3%)	11 (64.7%)	0.038^a^
Pre-op Lid fissure (mm)	4.27 ± 0.94 (2.50–6.00)	3.18 ± 0.90 (1.50–5.00)	< 0.001^b^
pre-op Lid fissure (mm) ≤5.00	28 (90.3%)	17 (100%)	0.543
pre-op Lid fissure (mm) ≤4.50	20 (64.5%)	16 (94.1%)	0.035^a^
Pre-op chin up			
yes	17 (54.8%)	14 (82.4%)	0.068
no	14 (45.2%)	3 (17.6%)	
Pre-op strabismus			
yes	5 (16.1%)	5 (29.4%)	0.295
no	26 (83.9%)	12 (70.6%)	
Prep-op amblyopia			
yes	14 (45.2%)	9 (52.9%)	0.766
no	17 (54.8%)	8 (47.1%)	

^1^No amblyopia in the final follow up

^2^Amblyopia in the final follow up

^3^Refraction unit = Diopter

^a^The *P* value was estimated by Fisher’s exact test

^b^The *P* value was estimated by student t test

Table 3 lists the various factors related to amblyopia at the final visit after surgery. At the final follow-up, postoperative astigmatism was significantly associated with final amblyopia (*p* = 0.037), whereas postoperative spherical equivalent and postoperative anisometropia were not associated with final amblyopia. MRD1 elevation > 3 mm and lid fissure elevation > 3.5 mm were associated with final amblyopia (*p* = 0.032 and *p* = 0.039, respectively). The results may be related to preoperative lid height. In order to achieve the desired postoperative lid height, patients with smaller preoperative MRD1 underwent greater MDR1 elevation, and those with lower preoperative lid fissure height underwent greater lid fissure height elevation. There was no correlation between type of sling used in surgery and final amblyopia. Of the 48 eyes, 39 (81.3%) had a good outcome, 8 (16.7%) had a moderate outcome, and 1 (2.0%) had a poor outcome. The above numbers were the sum of those with or without amblyopia. The cosmetic score was not associated with final amblyopia (Table 3).

**Table 3:** Factors related to the amblyopia in the final visit after surgery

Factor	No amblyopia ^1^	Amblyopia ^2^	*P* valve
	*n* = 31	*n* = 17	
Sling type			
mersilene mesh	24 (77.4%)	12 (70.6%)	0.878
silicone	4 (12.9%)	2 (11.8%)	
PTFE (Gore-Tex)	3 (9.7%)	3 (17.6%)	
Post-op sphere^3^	0.17 ± 1.21	0.66 ± 2.03	0.378
Post-op astigmatism^3^	− 1.15 ± 1.14	−1.97 ± 1.49	0.037^b^
Post-op spherical equivalent^3^	− 0.40 ± 1.35	−0.32 ± 1.73	0.874
Post-op anisometropia (astigmatism)^3^	1.01 ± 0.94	0.82 ± 0.88	0.511
Post-op anisometropia (spherical equivalent)^3^	0.96 ± 1.11	1.10 ± 1.12	0.680
Post-op MRD1 (mm)	2.21 ± 0.76	2.26 ± 0.69	0.806
MRD1 elevation (mm)	2.48 ± 1.14	3.50 ± 1.85	0.023^b^
MRD1 elevation (mm) > 2.5	12 (38.7%)	10 (58.8%)	0.232
MRD1 elevation (mm) > 3.0	8 (25.8%)	10 (58.8%)	0.032^a^
MRD1 elevation (mm) > 3.5	5 (16.1%)	10 (58.8%)	0.004^a^
Post-op Lid fissure (mm)	7.05 ± 0.86	6.74 ± 0.95	0.252
Lid fissure elevation (mm)	2.77 ± 1.03	3.56 ± 1.17	0.020^b^
lid fissure elevation (mm) > 2.5	15 (48.4%)	12 (70.6%)	0.224
lid fissure elevation (mm) > 3.0	11 (35.5%)	10 (58.8%)	0.140
lid fissure elevation (mm) > 3.5	5 (16.1%)	8 (47.1%)	0.039^a^
Post-op lago (mm)	1.31 ± 0.64	1.29 ± 0.53	0.946
Cosmetic score			
good^4^	25 (80.6%)	14 (82.4%)	1.000
Moderate^5^	5 (16.1%)	3 (17.6%)	
Poor^6^	1 (3.2%)	0 (0%)	

^1^No amblyopia in the final follow up

^2^Amblyopia in the final follow up

^3^Refraction unit = Diopter

^4^Post op MRD1 ≥ 2 mm or bilateral nonsymteric ≤1 mm

^5^Post op 2 mm > MRD1 > 1 mm or bilateral nonsymetric > 1 mm

^6^Post op MRD1 ≤ 1 mm or bilateral nonsymetric ≥2 mm

^a^The *P* value was estimated by Fisher’s exact test

^b^The P value was estimated by student t test

Table 4 lists the complications of surgery, which included sling exposure without infection in 1 Mersilene mesh case and exposure keratitis after 3 months in 4 Mersilene mesh cases and 1 polytetrafluoroethylene case. None of the cases developed sling infection. The incidence of sling exposure was 2.8%, and the incidence of exposure keratitis after 3 months was 10.4%. All cases of exposure keratitis were managed with lubricant medication without the need for revision surgery.

**Table 4:** Complications of surgery

Factor	Mersilene mesh	Silicone	PTFE (Gore-Tex)	Total
	*n* = 36	*n* = 6	*n* = 6	*n* = 48
Sling exposure	1 (2.8%)	0 (0%)	0 (0%)	1 (2.8%)
Sling infection	0 (0%)	0 (0%)	0 (0%)	0 (0%)
Exposure keratitis after 3 months	4 (11.1%)	0 (0%)	1 (16.7%)	5 (10.4%)

## Discussion

Congenital ptosis with poor levator function of less than 4 mm is usually corrected with frontalis suspension surgery. Frontalis suspension surgery can be performed using various techniques and different sling materials [13–15]. Materials used can be autogenous or banked fascia and alloplastic materials including silicone, Mersilene mesh, braided polyester, polypropylene, nylon, silk, collagen, stainless steel, and PTFE [13, 16–21]. Prior research has suggested that double slings to Crawford allow better results than thinner single slings [22]. Ben Simon and Goldberg reported no statistically significant difference in results between different suture materials or loop shape used in frontalis suspension surgery. In recent years, many studies have evaluated the functional success of various sling materials used in frontalis sling surgery [13, 23–25]. In the current study, frontalis suspension surgery was performed using Mersilene mesh, silicone rod, or PTFT, and no significant difference in surgical outcome or presence of amblyopia at the final follow-up was observed, further indicating that sling material is not related to final amblyopia.

Amblyopia has an estimated prevalence of 3.0 to 3.2% in the general population. Among patients with childhood ptosis, the incidence of amblyopia has been reported to be higher than that in the general population. [1, 2] Amblyopia with any form of childhood ptosis occurred in 14.9% of a cohort of 107 patients [7]. Of 96 patients in this cohort, 14% who were diagnosed with a congenital form of ptosis demonstrated amblyopia. These rates are at the low end of the range of previous non-population-based estimates, which were reported to be between 14 and 48% [1–3]. In our study, twenty three eyes (47.9%) were diagnosed with amblyopia prior to surgery, and 17 eyes (35.4%) had amblyopia at the final visit, suggesting that 6 eyes (12.5%) improved after undergoing both ptosis surgery and occlusion with or without refractive treatment. The incidence of amblyopia at the final follow-up was 35.4%, and this higher rate of amblyopia might be attributed to the greater severity in these cases due to the need for frontalis sling surgery.

The prevalence of amblyopia may correlate with the severity of ptosis [1, 5, 26]. The cause of the increased prevalence of amblyopia among patients with congenital ptosis remains debatable. Occlusion of the visual axis was found to be the leading cause of amblyopia in patients with congenital ptosis in prior research [7]. Subsequent studies have demonstrated that 1.6 to 12.3% of patients with congenital ptosis will have amblyopia due to occlusion of the pupil [1, 4, 5]. Prevalence of amblyopia has been previously reported to be as high as 56.4% in patients with blepharophimosis [27]. Hornblass et al. found a statistically significant correlation between severe nonocclusive ptosis (≥ 4 mm) and the development of amblyopia [26]. In this study, factors significantly associated with final amblyopia were blepharophimosis, preoperative MRD1 < − 1 mm, and preoperative lid fissure < 4.5 mm, all of which were related to severity of visual axis occlusion. Moreover, patients with smaller preoperative lid fissure were more likely to have amblyopia at the final visit. All patients diagnosed with amblyopia were treated with occlusion therapy and refraction error correction. Although patients with a poor or moderate cosmetic score experienced more lid drooping than those with a good cosmetic score during the follow-up period, cosmetic score was not related to amblyopia at the final visit. Final MRD1 and lid fissure showed no relation with amblyopia at the last visit, whereas preoperative MRD1 and lid fissure were indeed related to the incidence. Preoperative but not postoperative occlusion of the visual axis was a factor related to final amblyopia, even after frontalis sling surgery and amblyopia treatment.

Several large retrospective studies evaluating congenital ptosis revealed that the leading causes of amblyopia are strabismus and refractive errors [2–5]. Srinagesh et al. reported that almost all cases of congenital ptosis with amblyopia occur in the context of coexisting anisometropia or strabismus [3]. Harrad et al. also found that of 216 cases of simple congenital ptosis, 17% developed amblyopia, among which 21% had anisometropic amblyopia [4]. Stark et al. reported a 40% incidence of refractive errors causing amblyopia in congenital ptosis patients [28]. Oral et al. observed that the overall incidence of refractive errors causing amblyopia was much higher, with 71% of patients having congenital ptosis [2]. Schneider et al. reported that the incidence of astigmatism causing amblyopia was 28% in unilateral ptosis and 46% in bilateral ptosis [29]. In the present study, preoperative anisometropia (spherical equivalent) and postoperative astigmatism were significantly related to amblyopia at the final visit.

While the incidence of strabismus ranges between 1 and 5% in the general population, it has been reported to be 12 to 76% in patients with congenital ptosis [1, 2, 4, 5, 30]. Schneider et al. reported that amblyopia was related to strabismus in only 6% of patients; the rate of amblyopic refractive errors combined with strabismus was higher in eyes with severe ptosis [29]. In this study, preoperative strabismus was found in 16.1% of eyes without final amblyopia and in 29.4% of eyes with final amblyopia, implying that strabismus may be associated with amblyopia. However, further analysis of our data revealed that strabismus was not a significant factor related to amblyopia at the final visit after frontalis sling surgery.

This study has some limitations. First, the sample size was relatively small. In addition, the study was retrospective in design and preoperative refraction data were missing for 4 eyes of very young patients. To better elucidate the factors related to amblyopia in children with congenital ptosis after frontalis sling surgery, a study of larger scale involving a greater number of patients would be required. In particular, the numbers of cases using silicone rod and PTFT were small, and more cases for analysis would be desirable. Although the age at which the patients underwent frontalis sling surgery was not a factor related to amblyopia, the children with amblyopia at the last visit tended to receive the procedure at an earlier age in this study, which might be related to smaller preoperative lid fissure height. Those with smaller lid fissure height usually would undergo earlier frontalis sling surgery.

Despite these limitations, this study successfully identified factors related to amblyopia at the final visit through a review of cases of children with congenital ptosis undergoing frontalis sling surgery. All of the included patients underwent corrective surgery due to poor levator muscle function. This study found that Mersilene mesh, silicone rod, and PTFT can be safely and effectively used in frontalis sling surgery in children with no significant difference in incidence of final amblyopia among the materials used. Blepharophimosis, preoperative MRD1 ≤ − 1 mm, preoperative lid fissure ≤4.5 mm, preoperative anisometropia (spherical equivalent), and postoperative astigmatism were associated with amblyopia at the final visit. Results suggest that children with smaller lid fissure, higher preoperative anisometropia, and postoperative astigmatism may have a greater risk of amblyopia even after frontalis sling surgery. Furthermore, in the management of children with ptosis, amblyopia treatment for improving vision should not be overlooked, even after frontalis sling surgery.

## Abbreviations

logMAR: Logarithm of the minimum angle of resolution
MRD1: Margin reflex distance 1
PTFE: Polytetrafluoroethylene
